# The Synthesis of Polymeric Nanospheres and the Application as High-Temperature Nano-Plugging Agent in Water Based Drilling Fluid

**DOI:** 10.3389/fchem.2020.00247

**Published:** 2020-04-15

**Authors:** He Li, Kaihe Lv, Xianbin Huang, Zhen Lu, Xiaodong Dong

**Affiliations:** ^1^Key Laboratory of Unconventional Oil & Gas Development, China University of Petroleum (East China), Ministry of Education, Qingdao, China; ^2^School of Petroleum Engineering, China University of Petroleum (East China), Qingdao, China

**Keywords:** polymeric nanospheres, nano-plugging agent, high-temperature, water based drilling fluid, wellbore stability

## Abstract

Nanoscale plugging agent is essential to wellbore stability of troublesome shale formation in the drilling of oil and gas wells. In this paper, polymeric nanospheres (PNS) with a double cross-linked structure were synthesized using monomers of styrene (ST), acrylamide (AM), 2-Acrylamide- 2-methylpropanesulfonic acid (AMPS), and dimethyl diallyl ammonium chloride (DMDAAC). PNS were characterized by FTIR, SEM and TGA. The plugging performance of PNS was analyzed using nitrogen adsorption experiments and SEM. And compatibility with water based drilling fluid (WBM) was studied. Experimental results showed that PNS had a mean particle size of 133 nm, and could retain about half of the original size after high temperature treatment under 150–200°C. TGA showed that the initial decomposition temperature of PNS is around 315°C. After plugging by PNS, both the specific surface area and pore volume of the shale cuttings decreased substantially compared with those of shale samples treated with water. Thus, PNS was thermal stable in WBM under high temperature and could effectively plug shale pores. Besides, PNS was beneficial to reduce both API and HTHP fluid loss of WBM.

## Introduction

Shale formations account for about 75% of drilled formations worldwide (Osisanya and Chenevert, 1994). The shale formation has a large amount of micropores (Alharthy et al., 2013, 2016). On the one hand, the invasion of drilling fluids into micropores during the drilling process causes hydration and expansion of clay minerals (Luo et al., 2017; Huang et al., 2018a). On the other hand, the pressure transmission (Chen et al., 2010; Sarout and Detournay, 2011) of drilling fluids into the shale formation reduces the differential pressure, which weakens the support role played by drilling fluids. The above two factors result in wellbore instability (Lv et al., 2020) during the drilling process, including wellbore collapse, stuck pipe, necking, etc., which seriously affects the safety and efficiency of drilling and causes huge economic losses. According to statistics, economic losses caused by wellbore instability are estimated $800 million each year worldwide (AL-Bazali, 2011).

Effective plugging of micropores is one of the key measures to solve the wellbore instability. For shale formations, microscale and nanoscale pores coexist (Jin J. et al., 2017) and the nanoscale pores occupy the majority proportion (Cao et al., 2015; Jin et al., 2019; Zhao et al., 2019). Pore size distribution analyzed by precise measurement of SEM, nitrogen adsorption, nuclear magnetic resonance (NMR) indicates that the size of nanopores is in a range of a few nanometers to a few hundred nanometers (Curtis et al., 2011; Cao et al., 2015; Jin X. et al., 2017; Zhao et al., 2019). For micron-level pores, the variety of plugging agents is abundant, which includes ultra-fine calcium carbonate, fibers, asphalts, polymers, gel particles. However, these micron-level plugging agents could not plug nanoscale pores because their size is too large. Considering matching of size and according to many studies (Taha and Lee, 2015; Xu et al., 2017; Huang et al., 2018b), the nanoscale plugging agent is essential to wellbore stability of troublesome formation.

Aiming to solve the wellbore instability, inorganic nanomaterials such as nanosilica, nano CaCO_3_ were firstly used to formulate nano drilling fluid. Cai et al. (2012) found that adding unmodified nanosilica into WBMs could significantly reduce the invasion of water into the shale. Some research studied the effect of other inorganic nanoparticles on the wellbore stabilizing performance of drilling fluid such as nano grapheme (Taha and Lee, 2015), nano TiO_2_ (Cheraghian et al., 2014), and nano MgO (Alssafar and Al-Mahdawi, 2019). Nano polymers (Xu et al., 2017, 2018; Zhang et al., 2018) having good flexibility could plug irregular shale pores by transformation and have strong interaction with shale matrix. Polymer/inorganic nano composites (Wu et al., 2016; Huang et al., 2018b; Qiu et al., 2018) combining the advantages of polymeric and inorganic plugging agents are another research hotspot. But the polymer matrix has limited high-temperature resistance performance, leading to bad plugging performances in deep formation. Severe decomposition of polymer matrix will occur at high temperatures. High-temperature hydrolysis (Ma et al., 2015) is the most important form of polymer degradation in WBM.

This paper introduced the synthesis of a kind of polymeric nanospheres—PNS. The double cross-linked structure largely improved the high-temperature performance in water based drilling fluid. Experimental results indicated that PNS developed in this paper had good high-temperature performance and plugging performance.

## Experimental Section

### Materials

Acrylamide (AM, 99 wt%), 2-Acrylamide-2-methylpropanesulfonic acid (AMPS, 99 wt%), styrene (ST, 99 wt%), dimethyl diallyl ammonium chloride (DMDAAC, 60 wt% water solution), N, N'- methylene bisacrylamide (MBA, 99 wt%), potassium peroxodisulfate (K_2_S_2_O_8_, 99.5wt%), sodium hydroxide (NaOH, 99.5 wt%), and (3-aminopropyl)-triethoxysilane (KH550, 99 wt%) were provided by Aladdin Reagent Co., Ltd. Bentonite and outcrop shale samples were provided by Greatwall Drilling Company, China. The mineral compositions of the shale sample analyzed by X-ray diffraction were shown in Table 1.

**Table 1:** Mineral Compositions of shale sample by X-ray diffraction analysis.

**Mineral compositions**		**Content (%)**
Quartz		34.0
K feldspar		9.0
Anorthose		20.0
Calcite		18.0
Clay minerals	Kaolinite	3.0
	Illite	9.0
	Chlorite	1.5
	Illite/Smectite	5.5
	(I/S) mixed layer	(I/S ratio is 50%)

### Structure Design Principles of PNS

**Scheme 1:**
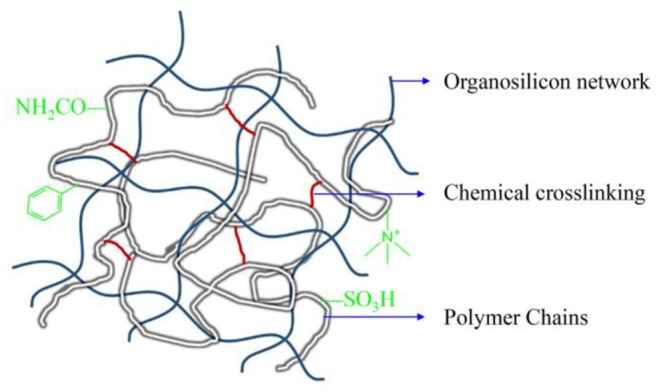
The double cross-linked structure of polymeric nanospheres (PNS).

1. PNS should have hydrophilic surfaces so that it could be easily dispersed into WBM.
2. Cationic monomer DMDAAC was used to enhance the attraction force between PNS and shale matrix through electrostatic interaction.
3. It has been well-studied that the use of the monomers of the ST and AMPS could increase the thermal stability of polymeric drilling fluid additives (Wu et al., 2001; Huo et al., 2018; Mohamadian et al., 2019) because the rigid side-chain of ST and hydrolysis resistant group (—SO_3_H) of AMPS are beneficial to temperature resistant property of polymeric additives.
4. In order to further improve the thermal stability of PNS in WBM, the cross-linking agent MBA and the silane coupling agent KH550 were used to form double cross-linked structure to decrease the mobility of polymer chains. The double cross-linked structure of PNS was shown in Scheme 1.

### Synthesis of PNS

**Scheme 2:**
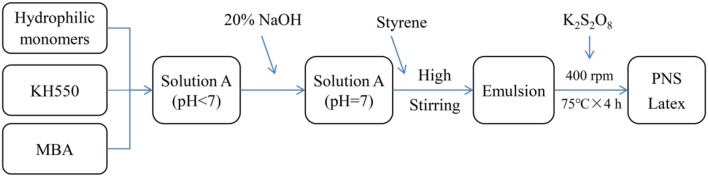
The synthesis process of polymeric nanospheres (PNS).

PNS were synthesized by the method of soap-free emulsion polymerization. The synthesis process of PNS is shown in Scheme 2. Five gram AMPS, 2 g AM, 3 g DMDAAC, 0.05 g KH550, and 0.1 g MBA were slowly added into a beaker with 150 mL DI water. The pH of the mixture was adjusted to 7.0 by adding 20 wt% NaOH solution. Fifty gram ST was then added into the mixture. And the mixture was emulsified using a high speed emulsifying machine and then transferred into a three-neck flask. After the emulsion in the flask was heated up to 75°C, 0.1 g K_2_S_2_O_8_ was added. The reaction was kept at 75°C for 4 h at a stirring speed of 400 rpm.

### Characterization of PNS

Two milliliter PNS latex was diluted by 50 times. Particle size distribution of PNS was measured using a Zetasizer Nano ZS90 laser particle size analyzer (Malvern, UK). A small drop of the diluted latex was dropped onto an anisotropic conductive film and was dried using an infrared light. Morphology of PNS on the film was observed with a scanning electron microscope (SEM, S-8200, Hitachi, Japan).

The solid sample of PNS was obtained by drying the PNS latex using a drying oven at 105°C and ground into powder in an agate mortar. The FTIR spectrum of the PNS powder sample was obtained using an IRTracer-100 FTIR spectrometer (Shimadzu, Japan) at room temperature. The frequency range was from 400 to 4,000 cm^−1^ with a resolution of 4 cm^−1^. Thermal gravimetric analysis of the PNS powder sample was carried out by a TGA550 Mettler-Toledo instrument (METTLER TOLEDO, USA) under nitrogen circumstances. The temperature range was 40–100°C with a heating rate of 20°C/min.

### Plugging Performance

Nitrogen adsorption experiment can accurately quantify characteristics of shale pores. Shale cuttings with 6–10 mesh were used for this experiment. The plugging performance of PNS was evaluated by analyzing the changes in specific surface area, pore volume and pore size of shale cuttings before and after treatment.

The 10 g shale cuttings were soaked in 350 mL inhibitive solution in a suction flask for 4 h where the suction pressure was kept at 0.08 MPa. After that the cuttings were filtered out and dewatered at 102°C for 4 h. Nitrogen adsorption tests were conducted by a surface area and porosity analyzer (Autosorb-iQ, Quantachrome, USA). Before measurement, all the shale samples were degassed under vacuum at 300°C for 2 h. The Barrett–Joyner–Halenda (BJH) model and density functional theory (DFT) were used to analyze the specific surface area and pore volume.

A scanning electron microscope (SEM, S-8200, Hitachi, Japan) was employed to observe the changes in the microstructure of the cuttings before and after plugging.

### Influence of PNS on the Performance of Drilling Fluid

#### Rheological Performance

4 wt% base fluid was prepared by slowly dispersing 40 g bentonite into 1,000 mL DI water using an electric mixer and aged at room temperature for 24 h. The properties of the base fluids with and without 1 wt% of PNS were determined according to the American Petroleum Institute (API) Recommended Practice 13B-1 (API RP 13B-1, 2009): Recommended Practices for Field Testing Water-based Drilling Fluids. The apparent viscosity (AV) and plastic viscosity (PV) were measured using a ZNN-D6 six-speed viscometer (Qingdao Tongchun, China) under room temperature and calculated by Equations (1, 2).

(1) $$\begin{array}{r} \text{AV}=0.5\times \emptyset 600\text{ mPa s} \end{array}$$

(2) $$\begin{array}{r} \text{PV}=\emptyset 600-\emptyset 300\text{ mPa s} \end{array}$$

where ∅600 and ∅300 are the dial readings at 300 and 600 rpm, respectively.

#### API and High Temperature High Pressure (HTHP) Fluid Loss

API fluid loss volumes of drilling fluids were determined by a ZNS-2 type pressure filter (Qingdao Tongchun, China) at 0.69 MPa under room temperature.

The drilling fluids were aged at 150 and 180°C for 16 h in a hot rolling oven. After that the HTHP fluid loss volumes were determined by a GGS71-B type HTHP pressure filter (Qingdao Tongchun, China) at 150 and 180°C under a differential pressure of 3.5 MPa.

## Results and Discussion

### FTIR

**Figure 1:**
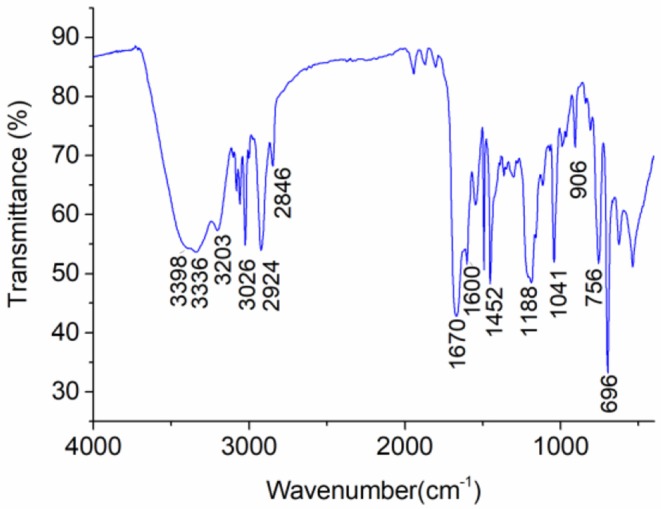
FTIR spectrum of synthesized polymeric nanospheres (PNS).

Figure 1 shows the FTIR spectrum of PNS. The peaks at 3,398 cm^−1^ and 3,203 cm^−1^ are attributed to the symmetric and anti-symmetric stretching vibrations of -NH_2_, respectively. The peak at 3,336 cm^−1^ is assigned to stretching vibration of N-H. The peak at 3,026 cm^−1^ is the characteristic peak of H-C=of the benzene ring. The peaks observed at 2,924 and 2,846 cm^−1^ are assigned to the symmetric and anti-symmetric stretching vibrations of C-H. The sharp peak at 1,670 cm^−1^ is the characteristic peak of C=O. The peaks at 1,600 and 1,452 cm^−1^ are the skeletal vibration of the benzene ring. The peaks at 1,188 and 1,041 cm^−1^ are the characteristic peaks of -${\text{SO}}_{3}^{-}$. The peak at 906 cm^−1^ is the characteristic peak of N-Cl from DMDAAC. The peaks at 756 and 696 cm^−1^ are assigned to out-plane vibration of C-H.

### Particle Size Distribution and SEM

**Figure 2:**
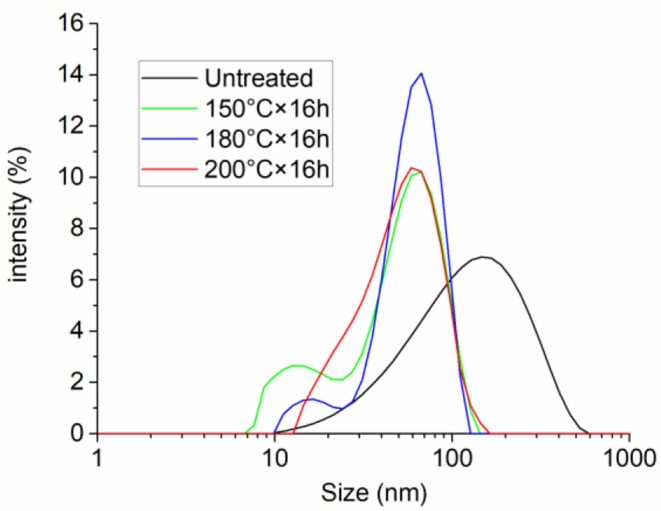
Particle size distribution of PNS and PNS after aging treatment at 150°C, 180°C and 200°C for 16 h.

**Figure 3:**
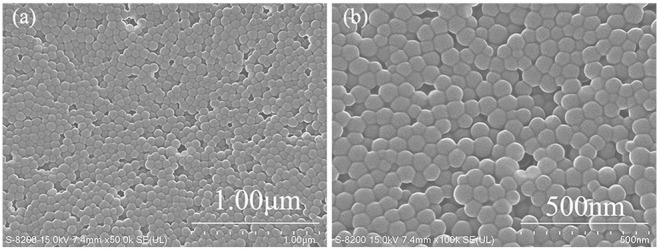
SEM images of PNS at different amplifications. **(a)** ×50K, **(b)** ×100K.

The particle size distribution analyzed by laser particle analyzer of PNS is shown in Figure 2. Experimental results showed that the particle size of the synthesized PNS was between 10 and 600 nm, and the mean particle size was 133 nm. Figure 3 shows the SEM images of PNS nanoparticles at different amplifications. It is apparent that PNS were nearly spherical flexible particles and their particle size was in accordance with the results obtained by the laser particle analyzer. In the synthesis process, ST is a hard monomer (Ouchi et al., 1981), which cannot form flexible polymers alone. The flexibility was obtained as a result of the addition of AM, AMPS, and DMDAAC. The flexibility enables PNS to deform and adapt to shale pores with a variety of shapes, which helps to increase plugging efficiency.

The particle size distributions of PNS latex after aging treatment at different temperatures for 16 h were also studied, as shown in Figure 2. The mean particle size was decreased to 64, 56, and 54 nm after treated at 150, 180, and 200°C, respectively. As the increase of aging temperature, the changes in mean particle size were small. It is concluded in this section that PNS could retain about half of the original size after high temperature treatment, which suggests that PNS could withstand high temperature in the water environment.

The high temperature resistance property in the water environment means that PNS had a strong hydrolysis resistance property under high temperature, the reason of which might come from two aspects. First, the amide groups and sulfonic acid groups of PNS molecules are hydrolysis-resistant chemical groups, which make PNS difficult to hydrolyze. Second, the double cross-linked structure improves the resistance to hydrolysis.

### TGA

**Figure 4:**
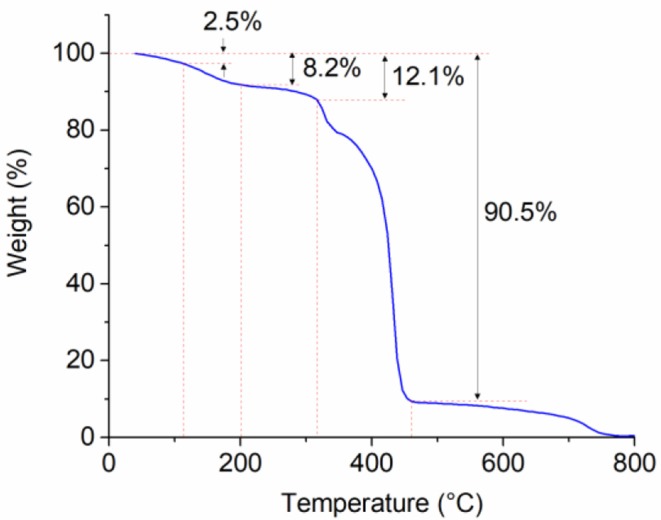
Thermogravimetric analysis of PNS under nitrogen environment.

The TGA curve of PNS is shown in Figure 4 where five weight loss stages are observed in total. The first stage is room temperature to about 110°C, which is caused by the volatilization of free water in PNS powder. The second weight loss stage between 110 and 200°C is possibly due to the break of a few amount of lateral groups such as amide groups, sulfonic acid groups, and the benzene ring groups. The third stage of 200–315°C is due to the thermal degradation of partial lateral groups (Chang et al., 2019). The sharp reduction in weight between 315 and 460°C is caused by the degradation of the polymer main chain (Liu and Yi, 2009). The last stage where temperature is above 460°C is caused by complex reactions, including the break of C-H and C-O.

The initial decomposition temperature of the polymer is around 315°C, which means the PNS could withstand high temperature and had a good thermal stability. The reason could be explained from two aspects. First, the benzene ring groups of PNS could increase the rigidity of the molecular chains and hinder the thermal movement of the molecular chain (Li et al., 2012), enhancing the thermal stability. Second, the double-crosslinked structure further restricts the movement of the molecular chains and thus enhances thermal stability (Mitomo et al., 2005).

### Plugging Performance

The plugging performance of PNS was analyzed in terms of specific surface area and pore volume of shale samples. The specific surface area and pore volume of shale pores analyzed by both BJH and DFT methods is shown in Table 2. In this section, original shale cuttings, shale cuttings treated with water and shale cuttings treated with 1 wt% PNS latex were analyzed.

**Table 2:** Pore analysis of original shale cuttings and shale cuttings treated with water and 1 wt% PNS latex determined by nitrogen adsorption experiments.

**Samples**	**Items**	**BJH model**		**DFT model**
		**Adsorption**	**Desorption**	
Original (non-treated)	Surface area (m^2^/g)	3.148	6.412	5.012
	Pore volume (cm^2^/g)	0.022	0.025	0.018
Treated with Water	Surface area (m^2^/g)	8.231	17.579	13.489
	Pore volume (cm^2^/g)	0.042	0.041	0.0345
Treated with 1% PNS latex	Surface area (m^2^/g)	4.16	9.74	7.458
	Pore volume (cm^2^/g)	0.029	0.034	0.021

The experimental results showed that the change law of specific surface area and pore volume are consistent for both models. The specific surface area and pore volume of the original shale cuttings were the smallest. After DI water treatment, both specific surface area and pore volume increased largely due to the hydration swelling of the shale cuttings. However, the drill cuttings plugged with 1 wt% PNS latex had a small increase in both specific surface area and pore volume compared to untreated drill cuttings. The above experimental phenomena showed that PNS is capable of plugging shale pores at a low concentration, but the specific surface area and pore volume still increase compared to the original state of shale due to the inevitable hydration swelling.

**Figure 5:**
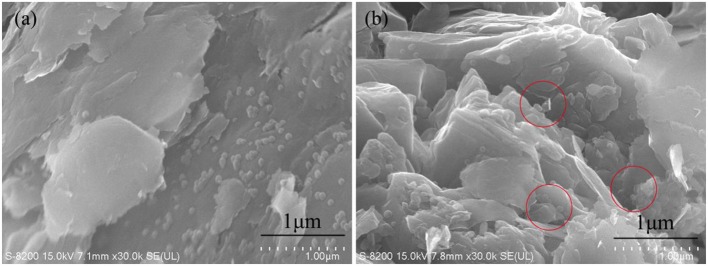
SEM images of shale cuttings plugged by 1 wt% PNS latex. Non-porous shale surface **(a)** and porous surface **(b)**.

The microstructure of the shale surfaces after plugged by 1 wt% PNS latex is shown in Figure 5. For the non-porous part of surfaces, PNS were uniformly adsorbed on the surfaces (Figure 5a). The easy adsorption on shale surfaces is the premise of effective plugging. And for surface areas with more pores, PNS were observed in the shale pores as was marked in Figure 5b. Thus, SEM proved the good plugging performance of PNS.

The easy adsorption of PNS on shale surfaces is one important reason for the good plugging performance. According to analysis of interaction forces, adsorption is formed through hydrogen bonding and electrostatic interaction. First, the surfaces of clay minerals have Si-OH and Al-OH, which could form hydrogen bonds with amide and sulfonic groups of PNS. Second, clay surfaces are permanently negatively charged. Because the monomer DMDAAC was used in the synthesis process, so the cations on PNS surfaces could establish electrostatic attraction forces. As a result, the two interactions forces enhanced the adsorption strength on the shale surfaces and plugging strength of shale pores.

Nano particles have a large specific surface area and are prone to aggregate, losing nanometer properties. Therefore, the nano-plugging agent must first have good dispersibility in water. In this paper, the hydrophilicity of the microspheres PNS is improved by adding hydrophilic monomers (AM, AMPS, DMDAAC), which is beneficial to the dispersion of microspheres.

### Influence of PNS on the Performance of Drilling Fluid

The application of PNS in drilling fluid must first ensure that PNS do not have a large negative impact on the rheological and filtration performance of drilling fluids. Table 3 shows the changes of rheology, API and HTHP fluid loss before and after adding 1 wt% PNS latex into the base fluid (4 wt% bentonite suspension). As is shown in Figure 3, the AV and PV changes of the base fluid can be ignored after the addition of 1 wt% PNS latex. And after aging at 150 and 180°C, the changes of AV and PV were very small compared with those of the base fluid aged at the same conditions. Therefore, PNS has almost no negative impact on the rheology of the drilling fluid.

**Table 3:** The influence of 1 wt% PNS latex on rheological and filtration performance of 4 wt% bentonite suspension.

**Fluids**	**Aging condition**	**AV (mPa·s)**	**PV (mPa·s)**	**FL_**API**_ (mL)**	**FL_**HTHP**_ (mL)**
Base fluid (4 wt% bentonite)	None	7.5	6	24	—
	150°C/16 h	7	5	29	78
	180°C/16 h	5	4.5	32.4	144
Base fluid + 1 wt% PNS latex	None	8	5	10.8	—
	150°C/16 h	6.5	5	17.6	56
	180°C/16 h	5.5	4	26.4	104

It also can be seen that after the addition of PNS, the API fluid loss decreased significantly. For the untreated base fluid, the API fluid loss was reduced from 24 to 10.8 mL after adding 1 wt% PNS latex. After high temperature aging at 150 and 180°C, the addition of PNS reduced API fluid loss to different extent. Besides, the addition of PNS also reduced HTHP fluid loss. Thus, PNS were beneficial to improve the filtration property of drilling fluids and had good thermal stability in WBM. It is worthy to note that filtration performance is different from plugging performance, which is because the pores of filter papers used in fluid loss measurements is micron-level. According to API standard (API RP 13B-1, 2009), the particle size retention range of these filter papers is 2–5 μm. While the range of shale pores varies from micron-scale to nanoscale, and majority of the pores are nanoscale.

## Conclusions

1. PNS were designed in chemical structure and synthesized by soap-free emulsion polymerization.
2. The particle size of PNS was between 10 and 600 nm with a mean value of 133 nm. The mean particle size was decreased to 64, 56, and 54 nm after aging at 150, 180, and 200°C for 16 h, respectively. TGA results showed that PNS had a high initial decomposition temperature of 315°C.
3. After plugging by 1 wt% PNS latex, both specific surface area and pore volume of the shale cuttings decreased substantially compared with those of shale samples treated with water, indicating PNS were effective to plug shale pores. SEM observation of shale cuttings proved the good plugging performance of PNS from the aspect of microstructure.
4. PNS was not detrimental to the rheology and filtration property of base fluid in the studied concentration. And PNS could reduce both API and HTHP fluid loss of base fluid.
5. PNS have a strong capability of resistant to hydrolysis and high temperature in WBM.

## Data Availability Statement

All datasets generated for this study are included in the article/supplementary material.

## Author Contributions

HL designed and carried out experiments. KL and ZL carried out experiments and analyzed the data. XH wrote the manuscript. XD helped perform the analysis and provided useful suggestions.

### Conflict of Interest

The authors declare that the research was conducted in the absence of any commercial or financial relationships that could be construed as a potential conflict of interest. The handling editor declared a shared affiliation, though no other collaboration, with the authors HL, KL, XH, ZL, and XD.
